# To Perform Thrombolysis or Not: A Case of Acute Pancreatitis Presenting with Chest Pain and Transient Left Bundle Branch Block

**DOI:** 10.1155/2010/204547

**Published:** 2010-09-14

**Authors:** S. Ullah, S. Mehmood, H. A. Chatha, A. Mahmood

**Affiliations:** Castle Hill Hospital, Cottingham HU16 5JQ, UK

## Abstract

A suspected case of acute coronary syndrome presented with new-onset left bundle branch and first-degree heart blocks. The decision to thrombolyse was reverted as ECG changes proved to be transient within fifteen minutes of presentation. Later on the patient was diagnosed with acute pancreatitis based on laboratory results of serum amylase, confirmed on radiological investigations.

## 1. Introduction

Chest pain is the most common presenting feature of ischemic heart disease and an estimated 300,000 people suffer from heart attack every year [1]. However, care should be taken while assessing these patients as other conditions may mimic a heart attack with corroborative ECG findings. We report a suspected case of acute coronary syndrome with new-onset left bundle branch block (LBBB), later diagnosed as acute pancreatitis.

## 2. Case Report

A 65-year-old man presented to accident and emergency (A&E) department with lower central chest and upper abdominal pain. The pain was constant, severe in intensity, dull in nature, and nonradiating. He felt nausea, shortness of breath and correlated this pain to previous attacks of angina pectoris. Past medical history and risk factors included hypertension, ischemic heart disease, and diabetes mellitus. 

On physical examination, the patient appeared pale, sweaty, stressed, and short of breath. Vital signs were recorded as pulse 80/min, blood pressure 110/60 mm Hg, respiratory rate 20/min, oxygen saturation 94% on air, and temperature 37.2°C. Cardiorespiratory examination revealed bilateral basal fine crepitations. An abdominal examination showed mild tenderness in the epigastrium with normal bowel sounds.

**Figure 1:**
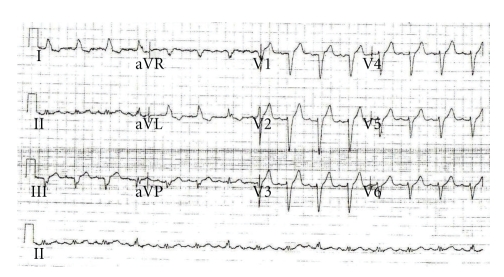
ECG immediately after the patient attended to A&E.

**Figure 2:**
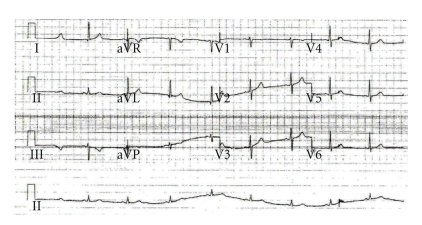
ECG fifteen minutes after the patient attended to A&E.

Two electrocardiograms (ECGs) recorded in 5 minutes interval demonstrated LBBB with first-degree heart block (Figure 1). The LBBB in his case was a new abnormality compared with old ECGs available in hospital notes. A diagnosis of Acute Coronary Syndrome (ACS) was made and thrombolytic therapy was planned in keeping with the hospital policy (door-to-needle within 30 minutes). The patient received initial treatment with oxygen, aspirin, glyceryl trinitrate spray, and intravenous diamorphine with little benefit. A 3rd ECG was recorded just before the commencement of thrombolytic therapy (Figure 2), which demonstrated normal sinus rhythm and complete resolution of 1st-degree heart block and LBBB. Further serial ECGs were consistent with the 3rd ECG. In view of the resolution of ECG abnormalities, it was decided not to thrombolyse the patient and only low molecular weight heparin was administered.

The results of laboratory investigations sent upon arrival in A&E department were available after 2 hours. The results revealed elevated levels of serum amylase 1210 U/L (normal range: 23–85 U/L) with normal liver function and renal function tests. Normal serum biochemical analysis ruled out any electrolyte abnormality to account for ECG changes [(Sodium 138 mmol/L (normal range: 135–146 mmol/L), Potassium 3.9 mmol/L (normal range: 3.5–4.8 mmol/L), Chloride 103 mmol/L (normal range: 99–109 mmol/L, Bicarbonate 28 mmol/L (normal range: 22–32 mmol/L, and Calcium 2.21 mmol/L (normal range: 2.12–2.63 mmol/L)]. A full blood count revealed high white cell count (WCC) 13.4 × 10^9^/L (normal range: 4.0–11.0 × 10^9^/L). Arterial blood gas analysis was within normal range. 

A diagnosis of acute pancreatitis was established, and a subsequent abdominal ultrasound scan confirmed findings suggestive of acute pancreatitis. Of note, serum Troponin-T levels at 8 hours and 12 hours post chest pain were not elevated. The patient did not undergo any further cardiac investigations, and his normal ECG remained unchanged for the course of hospital stay. He was conservatively managed for acute pancreatitis with an uneventful hospital stay and was discharged after 7 days.

## 3. Discussion

Acute coronary syndrome is one of the leading causes of deaths worldwide, and some 110,000 people succumb to it each year in the United Kingdom [1]. A person with symptoms of heart attack should be treated as soon as possible and thrombolytic treatment instigated within 60 minutes of call for professional help [1]. Primary percutaneous coronary intervention (PCI) is the treatment of choice for patients with ST segment elevation myocardial infarction (STEMI) [2]. Patients presenting with new-onset LBBB and ACS carry high mortality, and this group of patients benefits the greatest from early thrombolytic therapy or PCI [3]. Some centres practise PCI, and others still use thrombolytic therapy as main treatment modality based on the availability of the service.

The decision to initiate thrombolysis or PCI is usually based on history, examination, and ECG findings as most of the patients present well before the elevated levels of cardiac enzymes and troponins are measureable. Cardiac troponins are specific to heart muscle and can reliably detect heart muscle injury [4], however, a peak in their levels is noticed as early as 8 hours post myocardial injury, hence were assayed 8–12 hours after the onset of chest pain. 

Initial diagnosis of ACS in our case was based on clinical assessment and ECG abnormalities (lower central chest-to-epigastric pain, new-onset LBBB, 1st-degree heart block) which dictated thrombolytic therapy. Troponin assay was not requested upon presentation in A&E as the duration of pain was only 5 hours. However, the ECG changes proved to be transient and posed a diagnostic dilemma. The most probable explanation of transient ECG changes in this case is transdiaphragmatic epicardial irritation secondary to early acute pancreatitis. Absence of usual clinical symptoms and signs of acute pancreatitis (severe abdominal pain, tenderness, and guarding) in the presence of lower central chest pain resulted in initial consideration of ACS as the likely diagnosis.

Noncardiac causes of ECG changes secondary to abdominal pathologies (acute cholecystitis, pancreatitis, hepatitis, and peritonitis) have been reported in the literature [5–7]. Similarly, intrathoracic pathologies (aortic aneurysm, pneumothorax, and haemothorax), and intracranial causes (subarachnoid haemorrhage, thromboembolism, and cerebral oedema), may also cause ECG changes [8–10]. Whereas the presentation of acute pancreatitis with ECG changes has previously been reported [5, 11], to our knowledge such a peculiar presentation of transient LBBB and 1st-degree heart block with acute pancreatitis has not been reported. Clinicians need to be aware of such a peculiar presentation of acute pancreatitis. The challenge faced was to decide about potentially life saving thrombolytic treatment in view of changing ECG findings. A diagnostic elevation in amylase with supportive radiological appearance, however, did clarify the situation, but a patient with a true coronary event may not survive until those become available. 

It is important, however, to appreciate the limitation of this case when deriving conclusion. Whereas the diagnosis of acute pancreatitis was established with biochemical and radiological evidence in this presentation, the exclusion of ACS was based on cardiac troponin assays alone and coronary angiography was not performed. Due to the fact that more definitive cardiac evaluation was not performed, we cannot reliably exclude a coronary/cardiac cause of the LBBB. It is possible that the LBBB pattern could have been related to a coexistent ACS or other cardiac pathologies. Therefore, the findings of this paper should be considered within the context of its limitations. We recommend a detailed cardiac assessment in such cases to provide more accuracy in ruling out a coexistent cardiac pathology. Nevertheless, background knowledge of this association will enable clinicians to adopt an open approach in the assessment of such patients. It is vital that such cases be assessed and managed on an individual basis given the implications of misdiagnosis.

## 4. Conclusion

 An open approach, with repeated clinical assessments and serial ECGs, should be adopted in patients presenting with chest pain. Acute pancreatitis may present with symptoms mimicking acute coronary syndrome, and serum amylase assay, along with abdominal CT scan, may help establish early diagnosis whenever transient ECG findings are encountered.

##  Competing Interests 

The authors declare no competing interest**s**.
